# Maternal lipid profile and the relation with spontaneous preterm delivery: a systematic review

**DOI:** 10.1007/s00404-016-4216-5

**Published:** 2016-11-02

**Authors:** Maryam Moayeri, Karst Y. Heida, Arie Franx, Wilko Spiering, Monique W. M. de Laat, Martijn A. Oudijk

**Affiliations:** 1 Department of Obstetrics and Gynaecology, University Medical Center Utrecht, Utrecht, The Netherlands; 2 Julius Center for Health Sciences and Primary Care, University Medical Center Utrecht, Utrecht, The Netherlands; 3 Department of Vascular Medicine, University Medical Center Utrecht, Utrecht, The Netherlands; 4 Department of Obstetrics and Gynaecology, Academic Medical Center, H4-275, P.O.Box 22660, 1100 DD Amsterdam, The Netherlands

**Keywords:** Cholesterol, Homocysteine, Lipids, Preterm birth, Preterm delivery, Triglycerides

## Abstract

**Background:**

It is unknown whether an unfavorable (atherogenic) lipid profile and homocysteine level, which could supersede clinical cardiovascular disease, is also associated with an increased risk of spontaneous preterm delivery (sPTD). A systematic review of studies assessing the lipid profile and homocysteine value of women with sPTD compared to women with term delivery in pre-pregnancy and during pregnancy.

**Methods:**

A systematic search of peer-reviewed articles published between January 1980 and May 2014 was performed using MEDLINE, EMBASE and the Cochrane database. We included case–control and cohort studies that examined triglycerides, high/low density lipoprotein cholesterol, total cholesterol and homocysteine in women with sPTD. Articles were subdivided in pre-pregnancy, first, second and third trimester. Of 708 articles reviewed for eligibility, 14 met our inclusion criteria.

**Results and conclusion:**

Nine cohort studies and five case–control studies were analyzed, reporting on 1466 cases with sPTD and 11296 controls with term delivery. The studies suggest a possible elevated risk of sPTD in woman with high TG levels, no association of high and low density lipoprotein cholesterol with the risk of sPTD was found. High homocysteine levels are associated with sPTD in the second trimester. The role of triglycerides and homocysteine in sPTD should be explored further.

## Introduction

Preterm delivery is defined as delivery before 37 weeks of gestation [1]. Approximately 70 % of all PTDs are the result of spontaneous labour or preterm premature rupture of membranes (PPROM). Deliveries that follow spontaneous preterm labour and PPROM—together called spontaneous preterm deliveries (sPTD)—are regarded as a syndrome resulting from multiple causes, including infection or inflammation, vascular disease and uterine over distension [2]. Pathogenesis of this inappropriate early activation of uterine contractions and/or PPROM are not well understood. Although the precise mechanism cannot be established in most cases, some factors (e.g., previous preterm delivery, black race, smoking, advanced age, periodontal disease and low body mass index) have been pointed as high risk factors for sPTD. However, none of these factors include causal pathways to explain sPTD [2, 3].

Recently, it has been indicated that sPTD and cardiovascular disease share common risk factors [4]. Epidemiological evidence suggests that women who deliver preterm infants have a twofold increased risk later in life to develop cardiovascular disease [5]. In at least one third of women who deliver prematurely, vascular pathology is found in placentas [6]. Biopsies taken from placentas of women with spontaneous preterm labour showed histopathological ischemic changes such as villous infarcts, fibrinoid atherosis and thrombosis [6]. It is well established that an atherogenic lipid profile and/or elevated homocysteine concentration measured in serum or plasma are a strong and independent risk factor for vascular disease [7, 8]. Also the association of early maternal hypertriglyceridemia with pregnancy-induced hypertension is recognized [9]. Whether maternal lipid composition and homocysteine are also risk factors for prediction of preterm delivery through their vascular effects is still under debate [10]. Defining the exact role of possible subclinical, but relevant maternal atherogenic lipid profile and elevated homocysteine level as risk factors of sPTD is a reasonable goal for several reasons. First, identification of risk factors might provide important insights into mechanisms leading to sPTD. Second, identification of women at risk allows initiation of risk-specific treatment and tailored care during pregnancy. Third, the maternal lipid composition is a potentially modifiable risk factor to reduce the chance of recurrent PTD in a subsequent pregnancy and might define a population useful for studying specific interventions. For this review, we hypothesized that abnormal levels of lipids (i.e., total cholesterol, high or low density lipoprotein cholesterol, triglycerides) and homocysteine are associated with sPTD. To investigate this, we conducted a systematic review of existing literature.

## Methods

### Sources

**Fig. 1:**
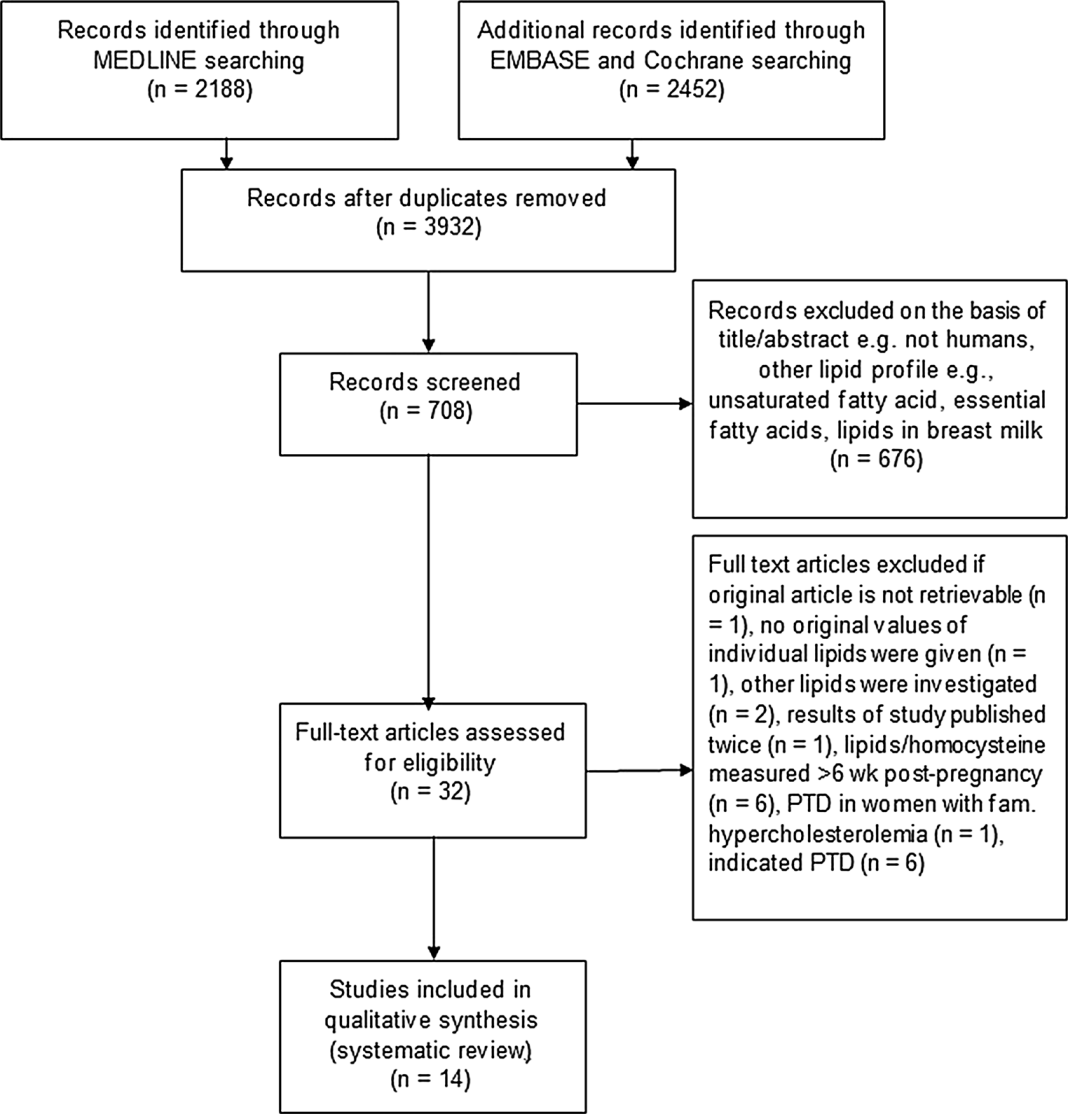
Flowchart of the literature reviewing process

This systematic review was performed according to the Meta-analysis of Observational Studies in Epidemiology (MOSE) guidelines. PubMed (MEDLINE), Embase and the Cochrane database were searched from January 1980 to May 2014 (by M.M.). The search strategy considered only articles written in English. Our search included combinations of the following terms in title or abstract: (premature delivery OR preterm birth OR preterm delivery OR premature birth OR “premature birth”[MeSH Terms]) AND (lipids OR “lipids”[MeSH Terms] OR HDL OR High Density Lipoprotein OR LDL OR low Density Lipoprotein OR homocysteine OR “homocysteine”[MeSH Terms] OR triglycerides OR “triglycerides”[MeSH Terms] OR cholesterol OR “cholesterol”[MeSH Terms]). Reference lists from included articles were also searched for additional eligible citations. The detailed workout of inclusion and exclusion criteria is listed in Fig. 1. Two authors (MM, KH) independently screened titles and abstracts of all retrieved studies. Full-text articles were obtained to assess the eligibility. In the case of disagreement, an additional author (MO) acted as arbitrator. Data extraction was performed by one author (MM) and was verified by another (KH). Quality assessment of each study was performed using the Newcastle—Ottawa Quality Assessment Scale and ranged between excellent quality graded 9 and poor quality graded 0 [11] (see Appendix Table 4).

### Study selection

We included cohort and case–control studies assessing the relation between lipid profiles measured pre-pregnancy or during pregnancy and sPTD. Control subjects had to be women with a term delivery.

Determinants evaluated were triglycerides (TG), high density lipoprotein cholesterol (HDL-c), low density lipoprotein cholesterol (LDL-c), total cholesterol (TC) or homocysteine (Hct). We divided these lipid profiles in period of measurement: pre-pregnancy and during the first, second or third trimester of pregnancy.

### Data extraction and analysis

Baseline characteristics of women with sPTD were compared to women with term delivery. For each included study the following characteristics were extracted: study design; characteristics of inclusion and exclusion criteria including sample size of cases; gestational age at sampling; gestational age at delivery; state of blood sampling, lipids/Hct levels and the covariates used to adjust the results. Lipids/Hct outcomes were reported as continuous values if available or odds ratios (OR). For consistency all units have been converted to mg/dL. If available the calculated relative risks or odds ratio’s in studies were evaluated in this review. However, some studies reported only mean values, for comprehensive purpose these results are also included in this review. No summary estimates of risks were calculated due to the different outcomes used in the studies and various periods in which the lipids were measured during pregnancy.

## Results

Systematic literature search of MEDLINE, Embase and Cochrane retrieved 708 articles, from which 32 were selected based on relevance and inclusion/exclusion criteria (Fig. 1). We included only original articles since no systematic reviews and meta-analyses were found. No additional relevant articles were identified after cross-check of the reference lists of the 32 articles. Full-text screening of the aforementioned articles resulted in exclusion of an additional 18 articles, resulting in 14 articles for final analysis.

### Characteristics of the studies

Extended baseline characteristics of the included studies are shown in Table 1. Nine studies were cohort studies, whereas five studies were case–control, reporting on a total of 1466 (range 40–221) cases with sPTD and a total of 11296 (range 50–4718) controls with term delivery. Nine articles measured lipid profile values during pregnancy [12–19], and three measured lipid profile values prior to pregnancy [20–22]. Three studies measured Hct values during pregnancy [23–25]. All studies used the same definition for preterm delivery (gestational age of <37 weeks) and term delivery (≥37 weeks of gestation). We considered a sPTD as spontaneous if this was exclusively mentioned in the article or when hypertensive disorders in pregnancy (preeclampsia, chronic- and gestational hypertension) were excluded during analyses. For a detailed list of the employed in/exclusion criteria in each study we refer to the Appendix Table 4.

**Table 1:** Characteristics of included studies

Study	Study design	sPTD (*n*)	Control (*n*)	Range of GA at sampling (wk)	Mean GA at sampling (wk)	GA at delivery (wk)		Maternal age at sampling (yrs)		BMI at sampling (kg/m^2^)		State of blood sampling	Biochemical factor(s) determined
						sPTD	Control	sPTD	Control	sPTD	Control		
Harville et al. [21], Finland	Retro. Cohort	67	67	–	384.8 ptp	–	39.8 ± 1.9	–		–	–	F	TC, TG, HDL-c, LDL-c
Catov et al. [20], USA	Prosp. Cohort	72	792	–	312 ptp	–	–	23.7 ± 3.8	24.2 ± 3.7	23.6 ± 4.9	23.4 ± 4.8	F	TC, TG, HDL-c, LDL-c
Magnussen et al. [22], Norway	Prosp. Cohort	172	4718	–	208 ptp	–	39.9 ± 2.1	26.3 ± 4.4^b^		24.4 ± 3.9^b^		NF	TC, TG, HDL-c
Chatzi et al. [13], Greece	Prosp. Cohort	45	145	<15	12.0 ± 1.5	–	–	29.45 ± 0.2^b^		–	–	F	TG, HDL-c
Vrijkotte et al. [14], The Netherlands	Prosp. Cohort	144	3912	<13	–	–	–	–	–	–	–	NF	TG
Catov et al. [12], USA	Case–control	90	199	<15	8.4 ± 2.5	–	–	24.6 ± 5.5	25.2 ± 6.1	25.6 ± 6.7	26.7 ± 6.3	NF	TC, TG, HDL-c, LDL-c
Edison et al. [18], USA	Retro. Cohort	70	1058	13–23	17.6 ± 1.5	–	–	–	–	–	–	–	TC
Alleman et al. [19], USA	Prosp. Cohort	153	2499	<26	–	33.7 ± 3.6	39.1 ± 1.1	–	–	–	–	NF	TC, TG, HDL-c, LDL-c
Mudd et al. [15], USA	Prosp. Cohort	221	988	15–27	–	–	–	–	–	–	–	NF	TC, TG, HDL-c, LDL-c
Niromanesh et al. [16], Iran	Prosp. Cohort	19	161	16–20	–	–	38.5 ± 2.1	28.1 ± 4.5		23.2 ± 1.3^b^		F	TG
Kramer et al. [25], Canada	Case–control	207	444	24–26	–	–	–	–	–	–	–	NF	TC, HDL-c, LDL-c, Hct
Knudtson et al. [24], USA^a^	Case–control	99	99	24–32	–	29.1 ± 2.9	39.4 ± 1.3	25 ± 6	24 ± 6	–	–	–	Hct
Bartha et al. [17], Spain	Case–control	40	50	24–36	31.3 ± 2.1	33.9 ± 2.6	39.6 ± 2.3	27.7 ± 6.5	29.6 ± 6.5	21.7 ± 3.0	23.6 ± 3.8	F	TC, TG, HDL-c, LDL-c
Dhoble et al. [23], India	Case–control	67	76	During delivery	–	39.3 ± 1.1	34.3 ± 2.2	23.0 ± 3.1	22.6 ± 3.0	20.1 ± 3.1	22.0 ± 2.9	–	Hct

Data are expressed as mean ± SD mg/dL

*Wk* weeks, *yrs* years, *sPTD* women with spontaneous preterm delivery, *control* subjects with term delivery, *GA* gestational age, *BMI* body mass index, *TC* total cholesterol, *TG* triglycerides, *HDL*-*c* high density lipoprotein cholesterol, *LDL-c* low density lipoprotein cholesterol, *Hct* homocysteine, *F* fasting, *NF* non-fasting, *Retr*. retrospective, *Prosp*. prospective, *Ptp* prior to pregnancy

^a^Knudtson et al. included only women with premature prelabour rupture of membranes. ^b^ Not stratified between cases and controls

Mean values of each lipid marker including Hct levels measured in women with sPTD and term delivery are shown in Table 2. More importantly, studies that reported the associated risk (odds ratio) of sPTD with the measured concentrations of lipids and Hct are shown in Table 3.

**Table 2:** Mean values of lipid profiles and homocysteine during pregnancy and delivery in woman with sPTD and controls

Period	Study	Case vs. control	Triglycerides	LDL-c	HDL-c	Homocysteine	Total cholesterol	Adjustment for confounders
First trimester	Alleman et al. [19]	sPTD	–	–	–	–	177.9 ± 35.7	Crude
		Control	–	–	–	–	173.8 ± 30.4	
	Catov et al. [12]	sPTD <34^#^	100.2 ± 60.2	118.3 ± 44.8	65.0 ± 18.7	–	*203.3* ± 50.5*	†
		sPTD 34–37^#^	*102.6* ± 43.8*	110.2 ± 37.9	65.7 ± 16.2	–	196.5 ± 43.7	
		Control	90.6 ± 41.5	104.7 ± 28.6	65.1 ± 16.2	–	188.0 ± 33.6	
Second trimester	Kramer et al. [25]	sPTD	–	116.0 ± 30.9	*61.9* ± 15.5*	*4.0* ± 1.4*	235.9 ± 42.5	‡
		Control	–	119.89 ± 30.9	69.6 ± 15.5	3.7 ± 0.9	232.0 ± 42.5	
	Mudd et al. [15]	sPTD	*171.1 (163.5*–179.0)*	70.3 (68.2–72.4)	116.4 (111.1–122.0)	–	*226.8 (220.9*–232.8)*	§
		Control	161.1 (157.3–164.9)	68.2 (67.2–69.2)	113.5 (111.0–116.0)	–	219.7 (217.1–222.4)	
Third trimester	Bartha et al. [17]	sPTD	189.4 ± 77.9	*125.7* ± 35.6*	*53.4* ± 18.2*	–	*219.6* ± 32.3*	–
		Control	175.0 ± 64.1	142.2 ± 36.1	68.3 ± 18.4	–	240.4 ± 40.0	
During delivery	Dhoble et al. [23]	sPTD	–	–	–	*12.2* ± 4.4*	–	–
		Control	–	–	–	10.7 ± 8.7	–	

Data are expressed as medians (range) or as means (SD) mg/dL

*sPTD* women with spontaneous preterm delivery, *Control* subjects with term delivery, *LDL-c* low density lipoprotein cholesterol, *HDL-c* high density lipoprotein cholesterol, *Reference* values expressed as mg/dL

^#^In weeks; * Significant at* p* < 0.05

^a^Gestational age at sampling, Body Mass-Index, race

^b^Maternal age, Body Mass-Index, smoking, socioeconomic status

^c^Maternal race, parity, gestational age at sampling

**Table 3:** Reported odds ratio or relative risk of woman with sPTD *vs.* controls during different sampling stages and gestational ages

Period	Study		Triglycerides (95 % CI)	LDL-c (95 % CI)	HDL-c (95 % CI)	Homocysteine (95 % CI)	Total cholesterol (95 % CI)	Adjustment for confounders
Pre-pregnancy	Harville et al. [21]	sPTD	0.98 (0.74–1.30)	1.17 (0.89–1.54)	0.92 (0.73–1.17)	–	1.13 (0.84–1.49)	†
	Catov et al. [20]	Q1	1.44 (0.78–2.67)	1.40(0.77–2.54)	0.98 (0.53–1.82)	–	1.66 (0.89–3.09)	‡
		Q2	Reference	Reference	Reference	–	Reference	
		Q3	1.41 (0.77–2.58)	1.05 (0.56–1.98)	0.72 (0.39–1.36)	–	1.57 (0.84–2.94)	
		Q4	1.02 (0.53–1.94)	1.30 (0.70–2.41)	1.32 (0.76–2.30)	–	1.55 (0.82–2.93)	
	Magnussen et al. [22]	0–20 %	Reference	–	1.4 (0.9–2.2)	–	Reference	§
		20–40 %	1.3 (0.8–2.1)	–	1.2 (0.7–1.9)	–	0.8 (0.5–1.3)	
		40–60 %	1.6 (1.0–2.5)	–	1.0 (0.7–1.8)	–	0.9 (0.6–1.4)	
		60–80 %	*2.1 (1.3*–3.2)*	–	1.1 (0.7–1.8)	–	1.1(0.7–1.6)	
		80–100 %	1.3 (0.8–2.2)	–	Reference	–	1.3 (0.9–2.0)	
First trimester	Alleman et al. [19]	Q1	1.12 (0.79–1.60)	1.02 (0.72–1.45)	1.16 (0.82–1.62}	–	0.90 (0.62–1.30)	Crude
		Q2–Q3	Reference	Reference	Reference	–	Reference	
		Q4	1.20 (0.85–1.69)	1.06 (0.75–1.51)	0.89 (0.62–1.29)	–	1.14 (0.81–1.61)	
	Vrijkotte et al. [14]	sPTD	0.87 (0.62–1.23)	–	–	–	–	||
	Chatzi et al. [13]	sPTD	1.09 (0.52–2.29)	–	1.54 (0.84–2.82)	–	–	¶
Second trimester	Alleman et al. [19]	Q1	1.10 (0.78–1.56)	1.25 (0.88–1.76)	1.21 (0.86–1.70)	–	1.00 (0.7–1.42)	Crude
		Q2-Q3	Reference	Reference	Reference	–	Reference	
		Q4	1.03 (0.72–1.47)	1.08 (0.76–1.54)	0.93 (0.64–1.34)	–	1.00 (0.7–1.42)	
	Edison et al. [18]	<10 %	–	–	–	–	*2.93 (1.54*–5.56)*	#
		10–90 %	–	–	–	–	Reference	
		≥90 %	–	–	–	–	*2.66 (1.39*–5.09)*	
	Kramer et al. [25]	Q1	–	Reference	Reference	Reference	Reference	**
		Q2	–	1.0 (0.5–2.2)	0.6 (0.3–1.2)	0.8 (0.4–1.4)	0.8 (0.4–1.7)	
		Q3	–	0.8 (0.4–1.8)	0.6 (0.3–1.2)	1.2 (0.7–2.0)	1.0 (0.4–2.2)	
		Q4	–	0.9 (0.4–2.0)	0.2 (0.1–1.6)	2.2 (1.3–3.7)	1.1 (0.5–2.3)	
	Mudd et al. [15]	<10 %	–	1.37 (0.85–2.21)	1.17 (0.70–1.95)	–	1.10 (0.67–1.82)	††
		10–70 %	–	Reference	Reference	–	Reference	
		≥70 %	–	1.17 (0.99–2.04)	1.10 (0.78–1.55)	–	1.51 (1.06–2.15)*	
		Q1	Reference	–	–	–	–	
		Q2	1.27 (0.81–2.01)	–	–	–	–	
		Q3	*1.90 (1.21*–2.97)*	–	–	–	–	
		Q4	*1.72 (1.06*–2.78)*	–	–	–	–	
	Niromanesh et al. [16]	sPTD	*10.9 (1.6*–74.4)*	–	–	–	–	Crude
Third trimester	Knudtson et al. [24]	>75 %	–	–	–	0.81 (0.4–1.6)		‡‡
		>90 %	–	–	–	1.7 (0.7–4.5)		
		>95 %	–	–	–	2.5 (0.7–8.7)		

Data are expressed as [quartile range = *Q*] or given percentage range and associated odds ratios or relative risks (95 % CI)

*sPTD* women with spontaneous preterm delivery, *Control* subjects with term delivery, *LDL-c* low density lipoprotein cholesterol, *HDL-c* high density lipoprotein cholesterol, *Reference* values expressed as mg/dL

* Statistically significant.^ †^ Race, parity, Body Mass-Index, physical activity at baseline, age, ever gestational hypertension or preeclampsia during follow-up.^ ‡^ Race, parity, BMI, physical activity at baseline, age, ever gestational hypertension or preeclampsia during follow-up. ^ §^ Maternal age at birth, parity, socioeconomic status, education, smoking, hypertensive disorders in pregnancy (preeclampsia, chronic. and gestational hypertension.^||^ Maternal age, ethnicity, parity, education level, pre-pregnancy Body Mass-Index, smoking during pregnancy, chronic hypertension.^ ¶^ Maternal age, education and smoking during pregnancy.^ #^ Age, maternal race, maternal weight, infant gender, presence of IUGR. ** Age, Body Mass-Index, smoking, socioeconomic status.^ ††^ Maternal race, parity, gestational age at sampling.^ ‡‡^ Age, smoking, presence of infection, low Body Mass-Index, nulliparity

**Table 4:** Appendix: Quality assessment of the reviewed studies and the in- and exclusion criteria used by the reviewed studies

**Study**	Ottawa Quality Assesment* *Selection/ Comparability/ Exposure*	**In- and exclusion criteria of study**
Alleman et al. [19], USA	3/0/3	In: PTD with spontaneous labour or PROM. Gestation age >20 weeks, singleton gestations
		Ex: congenital anomaly, any serious infection (gonorrhoea, syphils, hepatites), a term births with treatment tocolysis
Bartha et al. [17], Spain	2/0/3	In: threatened spontaneous PTD between 24 and 36 weeks gestational age, preterm labor was based on the clinical diagnosis of at least four painful uterine contractions per 20 minutes and evidence of cervical change
		Ex: maternal or fetal condition needing delivery (including symptoms or signs of chorioamnionitis), multiple gestations, PROM, intrauterine fetal demise or suspected lethal fetal anomalies, cervix dilated > 4cm, treatment with either tocolytics or corticosteroids within 24 hourspreviously, food intake within 8 hours previously
Catov et al. [12], USA	2/2/3	In: PTD with PROM, spontaneous labor
		Ex: hypertension (chronic, transient, PE), diabetes, growth restriction, small for gestational age (<10% percentile)
Catov et al. [20], USA	4/2/3	In: first birth with PTD< 37 weeks gestation (no distinction between indicated and spontaneous PTD)
		Ex: diabetes mellitus or hypertension at baseline based on self-report or medication use, pregnant within 3 months of the baseline assessment, hysterectomy at baseline, no births between baseline and yr 20 of follow-up, twin births, a first term birth but subsequent PTD
Chatzi et al. [13], Greece	4/1/2	In: PTD<37 weeks, singleton pregnancy,
		Ex: preeclampsia
Dhoble et al. [23], India	2/0/3	In: PTD, singleton pregnancy
		Ex: preeclampsia, gestational diabetes, anemia, chorioamnionitis, fetal infections, alcohol, drug abuse
Edison et al. [18], USA	3/1/3	In: PTD, aged 21 to 34
		Ex: history of smoking, type 1 diabetes, or other medical or gestational risk conditions
Harville et al. [21], Finland	3/2/3	In: PTD< 37 weeks, limited to primiparous women
		Ex: type 1 diabetes or suspected familial hypercholesterolemia, gestational hypertension, pre-eclampsia, gestational diabetes
Knudtson et al. [24], USA	3/2/2	In: PPROM, singleton pregnancy
Kramer et al. [25], Canada	2/2/3	In: PTD<37 weeks gestation with PPROM, age >18 years at the expected date of delivery, singleton gestation.
		Ex: severe chronic illness (other than hypertension, asthma or diabetes) requiring ongoing treatment, placenta previa, history of incompetent cervix diagnosed in a previous pregnancy, impending delivery at the time of initial contact or a fetus affected by a major anomely
Magnussen et al. [22], Norway	3/2/3	In: PTD as <37 weeks gestation, singleton pregnancy, at least 1 birth (gestational age 22 weeks or birthweight above 500 g, Ex: pregnant at baseline or delivered within 9 months after participating in, the study, hypertensive disorders in pregnancy (preeclampsia, chronic. and gestational hypertension
Mudd et al. [15], USA	2/0/2	In: Spontaneous preterm birth, singleton pregnancy, no known chromosomal abnormality or birth defect, maternal serum alpha-fetoprotein (MSAFP) screen at 15–22 weeks, maternal age≥15 years, no preexisting diabetes mellitus. All women with high MSAFP levels (i.e.≥2multiplesofthemean) were invited to participate because this biomarker has previously been associated with PTD.
		Ex: medical indicated PTD
Niromanesh et al. [16], Iran	2/0/2	In: PTD with age between 20–35 yrs, gravid >2
		Ex: history of PTD, pre-eclampsia, diabetes or gestational diabetes (GD), primigravida, those with a BMI >25
Vrijkotte et al. [14], The Netherlands	3/0/3	In: spontaneous PTD defined as delivery onset by spontaneous preterm labor or premature rupture of membranes between 24.0 and 36.6 wk gestation
		Ex: multiple gestation or who had no data on the gestational age at blood sampling, women with diabetes (preexistent as well as pregnancy induced), those using lipid-altering medication (*e.g.* antiepileptic drugs, steroids, insulin, antidepressants, thyroid hormones, or sleep medication),

### Pre-pregnancy

#### Total cholesterol (TC)

Three studies reported on the associated risk of sPTD with the measured TC concentrations in which 311 patients and 5577 control subjects were included [13–15]. Only one study, Catov et al., found both an increased risk of sPTD in the high and low levels of TC compared to the control group when stratified for gestational age <34 weeks [13] (Table 3).

### Triglycerides (TG)

Three studies reported on pre-pregnancy TG and the risk of sPTD in a total of 311 patients and 5577 control subjects [20–22]. The time interval between sampling and pregnancy ranged between 4 and 7.4 years. The results of two studies showed that the TG concentrations did not significantly influence the odds on sPTD [21, 26] (Table 3). The third study with the largest number of patients by Magnussen et al. reported a significantly increased relative risk of 2.1 (1.3–3.2) for sPTD in the 106–133 mg/dL TG range when compared to 18–53 mg/dL as reference range [22]. However, TG levels were measured in the non-fasting state and not adjusted for body mass index (BMI), which is a potentially important confounder. Remarkably, only the second highest TG levels were a significant risk factor, while the highest TG level was not (Table 3).

### High density lipoprotein cholesterol (HDL-c)

Three studies reported on HDL-c levels and the risk of sPTD counting a total of 311 patients and 5577 control subjects [20–22]. All three studies found no significant associations between sPTD and the measured HDL-c concentrations (Table 3).

### Low density lipoprotein cholesterol (LDL-c)

Two studies in which 139 patients and 859 control subjects were included reported on LDL-c levels and the risk of sPTD. Both studies did not report a significant association between sPTD and LDL-c levels [20, 21] (Table 3).

### During pregnancy

#### Total cholesterol (TC)

Six studies measured TC levels during pregnancy, in which 311 patients and 9489 control subjects were included [12, 15, 17–19, 25]. In the first trimester, two studies found no associations between TC levels and sPTD [12, 19]. Five studies analysed the association between TC concentrations in the sPTD group compared to the control group during second and third trimester, in which three found a significantly higher risk of sPTD in subjects with high TC levels [15, 17, 18], and one also found a higher risk of sPTD in subjects with low TC levels [18] (Table 3).

The fourth study by Catov et al. analysed the association between mean TC concentrations in the sPTD compared to the control group and found only a significantly elevated mean value of TC in the sPTD group after stratifying for a gestational age of 34–37 weeks [20].

#### Triglycerides (TG)

Seven studies reported on TG measurements during pregnancy, of which 712 patients and 4005 control subjects were included [12–17, 19]. Three out of four studies calculated the associated risk of sPTD with the measured TG concentrations in the first trimester and did not report any significant difference in risk [13, 15, 19]. The fourth study by Catov et al. analysed the association between mean TG concentrations in the sPTD compared to the control group and found only a significantly elevated mean value of TG in the sPTD group after stratifying for a gestational age of 34–37 weeks [27].

Four studies measured TG during second and third trimester [15–17, 19]. The first two studies calculated the risk of sPTD with the measured TG levels in the second trimester, of which only Niromanesh et al. found a significantly elevated relative risk 10.9 (1.6–74.4) in the >159 mg/dL TG range [16, 19]. Both studies did not adjust for confounders such as BMI. The second two studies analysed the association between mean TG concentrations in the sPTD compared to the control group of which only Mudd et al. found significant higher mean TG value in women with sPTD but without adjusting for BMI [15, 17] (Table 2).

#### High density lipoprotein cholesterol (HDL-c)

Six studies measured HDL-c levels during pregnancy, in which 756 patients and 8237 control subjects were included [12, 13, 15, 17, 19, 25]. Four studies reported no associations between sPTD and the measured HDL-c levels [12, 13, 19]. Kramer et al., however, found significantly lower mean HDL-c level in cases with sPTD [25]. During the late second to third trimester Bartha et al. found a significantly decreased mean level of HDL-c in cases with sPTD [17].

#### Low density lipoprotein cholesterol (LDL-c)

Five studies reported on LDL-c measurements during pregnancy in which 711 patients and 4180 control subjects were included. Only Alleman et al. analysed the relative risk of sPTD with the measured LDL-c levels during first and second trimester and observed no significant differences in risk [19] (Table 3). During late second to third trimester Bartha et al. found a significantly decreased level of mean LDL-c in the sPTD group [17].

#### Homocysteine (Hct)

Three studies measured Hct levels during pregnancy counting for 373 patients and 619 control subjects [23–25]. Kramer et al. reported a significantly higher odds ratio of 2.2 (1.3–3.7) on sPTD in the highest quartile compared to the lowest quartile (Table 3) Knudtson et al. reported no difference in mean Hct levels in the third trimester in cases versus controls [24]. Interestingly, Dhoble et al. found significantly higher values of Hct during delivery in patients with sPTD versus controls [23].

## Discussion

In this systematic review, data from 14 original articles were extracted to determine whether unfavorable lipid profiles and homocysteine values measured before and during pregnancy are associated with sPTD. The included studies showed considerable heterogeneity. However, our review was able to point out the following important findings.

### Total cholesterol

Total cholesterol (TC) is the sum of HDL-c, LDL-c, and VLDL-c, in which LDL-c and HDL-c levels—with opposite effects—are important in the decision whether treatment is necessary. This would reflect that TC level as an individual determinant for sPTD is clinically useless. However, all included studies in this review did measure and analyze TC, and therefore, we decided to include it in our review for comprehensive purpose.

All studies consistently report that pre-pregnancy TC levels are similar in sPTD and term delivery. This is also consistent with the findings of the Norwegian registry study in which no apparent increased risk of PTD was detected in woman with heterozygous familial hypercholesterolemia compared to the general population of woman of childbearing age [32]. Catov et al., however, suggest that early sPTD (<34 weeks) is associated with both high and low pre-pregnancy TC [18]. This same finding is supported by Edison et al. during second trimester pregnancy and by Mudd et al. only for high TC level [13, 25]. However, these findings could not be reproduced by Alleman et al. with similar study population and study characteristics [13, 17]. The pathogenesis leading to sPTD caused by low TC levels is likely caused by a distinct pathway and presumably differs from high TC levels. Low TC level is linked to poor nutritional status, which in general leads to a condition that is associated with adverse pregnancy outcomes including PTD [2, 18]. In addition, poor nutrition may enhance susceptibility to infection that is a known contributor to the pathogenesis of PTD [2]. High TC level on the other hand, may partially be explained by other risk factors, such as lifestyle and dietary habits. Several studies suggest that maternal BMI and low maternal socioeconomic status are related to the risk of PTD [2, 33, 34]. Most studies included in this review did not stratify for these known risk factors, limiting the interpretation of their results and conclusions.

#### Triglycerides

The analysed data on pre-pregnancy and pregnancy TG levels were highly heterogeneous. Yet, they showed a tendency towards no association between measured TG concentrations and the risk of sPTD. The exception to this conclusion are the findings of the two largest cohort studies performed by Mudd et al. and Magnussen et al, which suggested that high TG levels are associated with increased risk of sPTD. However, both studies did not adjust for pre-pregnancy BMI. Moreover, several studies suggest that maternal BMI is related to the risk of PTD [2, 28]. It is also important to refine their results as they used non-fasting venous blood samples, which may influence lipid levels. Although studies comparing fasting *vs.* non-fasting lipid levels show minimal differences (<5 %) for TC, HDL-c, and LDL-c values, TG may be affected by as high as 15 % in the non-fasted state [29]. This could potentially taint their results. However, in daily clinical practice it is challenging to obtain fasting blood samples from pregnant women. Therefore, it is likely not feasible in a general screening scenario.

#### High and low density lipoprotein cholesterol

The studies are mutually consistent that no associations between both HDL-c and LDL-c levels in pre-pregnancy and increased risk of sPTD could be found. However, inconsistent findings are reported during pregnancy. The studies that did report an increased risk such as Barthe et al. first: did not adjust the results for potential confounders such as maternal age, race, socioeconomic status and BMI; and second: graded poor on the Ottawa Quality Assessment Scale for study quality (see Appendix Table 4). Therefore, these results should be interpreted with caution.

#### Homocysteine

Only three studies that investigated the association between Hct and sPTD could be retrieved. Two of them suggest that a higher level of Hct during the second trimester and during delivery was associated with sPTD. These findings are potentially of great value to understand the mechanism leading to sPTD. The possible role of Hct in the pathogenesis of sPTB may be explained by the same role of Hct in the process of endothelial dysfunction leading to vascular pathology [8].

In general, the pathogenesis of sPTD is complex and in many cases, no distinct causal pattern is recognized. Though, it is well established that the risk of cardiovascular disease is higher in women who have experienced PTD or delivery of a small-for-gestational age infant [2, 4, 5]. Histopathologic similarities such as accelerated villous maturation and decidual arteriopathy in the placenta between preeclampsia, intrauterine growth restriction, and sPTD may suggest a common pathophysiological pathway. Therefore, we hypothesize that atherogenic lipid profile and homocysteine may induce atherosis in the uteroplacental spiral arteries. In PTD, these supplying blood vessels to the placenta appear to show failure of physiological transformation as seen in preeclampsia [30]. The latter could lead to placental ischemia and subsequently to decidual necrosis and hemorrhage at the uteroplacental interface resulting in premature contractions and/or PPROM [31], even in a normotensive pregnancy [25, 35]. These processes have been described in non-pregnant woman in which endothelial inflammation and infection induces changes in blood lipid levels and vice versa [36].

### Strengths and limitations

This is the first review that systematically addresses the relation between lipid profile/Hct and sPTD. However, we recognize several limitations. First, the strength of the review depends on the design and quality of the articles included (see Appendix Table 4). Dissimilarity in baseline data such as differences in lipid value cut-off levels in sPTD, which is essential for comparison of studies, precludes the collection of the results for meta-analyses.

Second, in most studies the maternal lipids or Hct were sampled only once during pregnancy, preventing us to describe the trajectory of lipid levels or compare pre-pregnancy and pregnancy levels in the same study. However, from the physiology of healthy term pregnancies we know that lipid markers and homocysteine are known to be affected by hormonal changes that occur in pregnancy.

Third, majority of studies did not adjust for the possible use of antenatal corticosteroids which is known to influence the lipid profile [36]. The use of antenatal corticosteroids is often a single high dose course to enhance fetal lung maturity in women with high risk of preterm delivery [38]. However, as all studies sampled during pre-pregnancy, first and second trimester confounding by the use of antenatal corticosteroids was unlikely. Only Bartha et al. and Vrijkotte et al. excluded women with corticosteroid use during pregnancy [14, 17]. Finally, extrapolation of these findings to the general population should be done with utmost caution. For future studies, general consensus should exist about generally accepted cut-off point and non-fasting state of blood samples. These studies may lead to recognition of one or more lipid markers with potential interest to a prediction model for sPTD. This prediction model should be first validated in the general population before lipids might be used as a screening tool to identify women at risk for sPTD.

## Conclusion

Our review suggests a possible elevated risk of sPTD in woman with high TG levels. However, due to inadequate adjustment for confounders such as BMI and non-fasting status, no definite conclusion could be drawn. We found no associations between HDL-c and LDL-c levels and sPTD. Limited data showed an association of higher levels of Hct with sPTD and could potentially be of clinical interest. Overall, these results support the need for a well-designed study exploring the possible clinical relevance of TG and Hct as biomarkers for prediction of sPTD.

## Appendix

See appendix table 4
